# CD19 chimeric antigen receptor T-cell therapy following autologous stem cell transplantation against relapsed or refractory Burkitt lymphoma/leukemia: A case report and literature review

**DOI:** 10.3389/fonc.2022.932254

**Published:** 2022-10-24

**Authors:** Mingyu Ye, Lei Gao, Tao Wang, Jiechen Yu, Jiaping Gui, Jianmin Yang

**Affiliations:** Department of Hematology, The First Affiliated Hospital of Naval Medical University, Shanghai, China

**Keywords:** Burkitt lymphoma, leukemia, CD19, chimeric antigen receptor T-cell, autologous stem cell transplantation

## Abstract

Burkitt lymphoma or leukemia (BL) is a highly aggressive non-Hodgkin lymphoma. Older age (over 60 years old) and the presence of high-risk factors (such as abdominal mass, high levels of the serum lactic dehydrogenase, Ann Arbor stage II-IV and so on) usually predict a poorer outcome. Chimeric antigen receptor T cells (CART) have achieved remarkable success in the treatment of B-cell leukemia and lymphoma. Here, for the first time, we report a 61-year-old, high-risk BL patient with autologous stem cell transplantation (ASCT) bridging therapy prior to CART as consolidation therapy. Our findings demonstrate that the combination of ASCT and CART for BL is safe and feasible.

## Introduction

Burkitt lymphoma/leukemia (BL) is a highly aggressive non-Hodgkin lymphoma characterized by rapidly progressive tumors with high extranodal involvement (1). It has been established that following dose-intensive chemotherapy, the three year-progression-free survival (PFS) and overall survival (OS) rates are 64 and 70%, respectively. However, the proportion of patients with BL who were over 60 years old was 24%, and the three-year PFS rate was only 56% (2). Age is an important predictor of outcome, as treatment-related mortality is high in older patients. For relapsed or refractory patients, high dose chemotherapy and autologous stem cell transplantation (ASCT) are recommended as salvage therapies.

In recent years, chimeric antigen receptor T cells (CART) have yielded unprecedented success with B-cell malignancies, with a complete remission (CR) rate of 81–93% (3, 4) in B-cell acute lymphoblastic leukemia and 40–59% in B-cell non-Hodgkin lymphoma (5–7). However, few studies have reported the potential of CART against BL.

The present case study is the first to report the treatment of a high-risk patient with BL with a combination of ASCT and CART therapy and the patient has now been in remission for 4 years. In this report, we describe the clinical course, including cytokine monitoring after CART infusion and the follow-up of expression of CD19 CAR T-cell expansion in peripheral blood. Our findings demonstrate that the combination of autologous stem cell transplantation and chimeric antigen receptor T-cells for Burkitt lymphoma is safe and feasible.

## Case report

A 61-year-old man exhibiting fever (38.5°C), bleeding gums, and pain in the upper abdomen was admitted to the Department of Hematology, Changhai Hospital on August 31, 2016. A complete blood count test revealed the following: white blood cell count: 27.25 × 10^9^/L, hemoglobin level: 139 g/L, and platelet count: 13 × 10^9^/L. Additional laboratory tests showed that the serum lactic dehydrogenase level was 2246 U/L (upper limit of normal: 310 U/L). The patient was diagnosed with BL by bone marrow aspiration and biopsy tests, with 79% lymphoma cells in the bone marrow. The cells expressed CD19, CD20, CD10, CD22, and CD38, and had a 47, XY,dup (1), t (8, 14)+18 (10)/46,XY (10) karyotype. Computed tomography revealed multiple enlarged lymph nodes in the neck, mediastinum, and retroperitoneum. Contrast magnetic resonance imaging revealed leukemia with multiple enlarged lymph nodes in the hilar area, mesenteric area, and retroperitoneum.

The patient received induction therapy with VDCP (vindesine, daunorubicin, cyclophosphamide, and dexamethasone) on September 1, 2016. Then, he was administered with six courses of alternative therapy with Hyper-CVAD-A (cyclophosphamide, vindesine, doxorubicin, and dexamethasone), MAVP (methotrexate, cytarabine, vindesine, and dexamethasone), and central nervous system prophylaxis. One month after the last round of therapy, B-ultrasound showed that the inguinal lymph nodes were enlarged; the largest was 2.3 × 0.6 cm.

**Figure 1:**
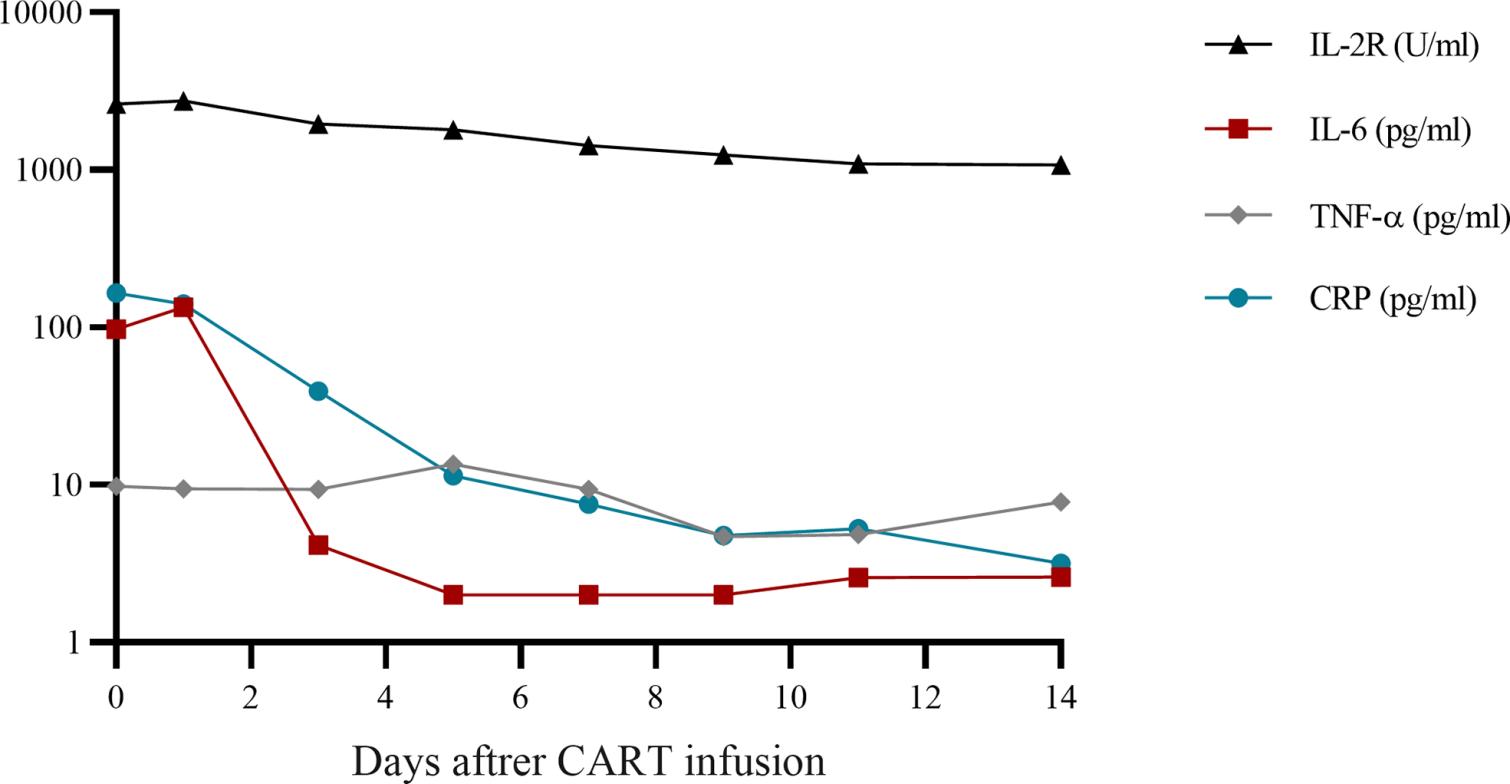
Cytokine monitoring after CART infusion. CART, chimeric antigen receptor T-cell; IL, interleukin; TNF, tumor necrosis factor; CRP, C-reactive protein.

**Figure 2:**
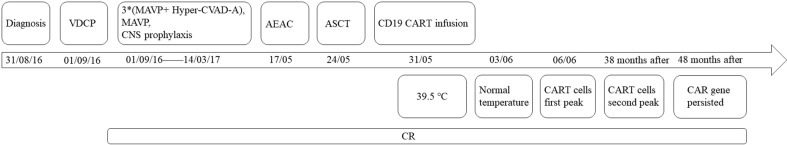
Timeline of treatment and efficacy. *multiply: six courses of alternative therapy with Hyper-CVAD-A (cyclophosphamide, vindesine, doxorubicin, and dexamethasone), MAVP (methotrexate, cytarabine, vindesine, and dexamethasone).

Therefore, the patient was enrolled in our clinical trials of ASCT bridging CD19 CART as consolidation therapies (NCT02672501). Peripheral blood lymphocyte separation and collection after a bone marrow aspiration biopsy showed complete remission, followed by CE regimen chemotherapy (cyclophosphamide 2g day1-2 and etoposide 330mg day1-2). When the patient’s blood routine level dropped to the lowest level, G-CSF (granulocyte-colony stimulating factor) 400ug were injected for 4 consecutive days. Peripheral blood mononuclear cells were collected using apheresis. The conditioning therapy for the patient undergoing ASCT was CEAC (semustine, etoposide, cytarabine, and cyclophosphamide), which included semustine (250 mg/m^2^) on day -6, cytarabine (500 mg/m^2^) every 12 h, and etoposide (300 mg/m^2^) and cyclophosphamide (1.0 g/m^2^) from days -5 to -2. The patient developed fever(37.8°C) and nasal congestion on day-4, and recovered after cefoperazone-sulbactam treatment. Autologous hematopoietic stem cells were infused on day 0 with a mononuclear cell dose of 4.2 × 10^8^/kg and a CD34^+^ cell dose of 2.69 × 10^6^/kg. On day +5, he developed fever(38.2°C) due to agranulocytosis, which returned to normal after 2 days of cefoperazone-sulbactam treatment. Additionally, 6 × 10^6^/kg CART cells were infused 7 d after ASCT. The CART products included 32.8% CD45^+^CD62L^+^ naive CART cells, 44.7% CD45RA^-^CD62L^+^ central memory CART cells, and 15.9% CD45RA^-^CD62L^-^ effector memory CART cells. Grade 1 cytokine release syndrome was reached according to the Penn grading scale, because the patient developed a high fever with a body temperature of 39°C 2 h after infusion (8). Vancomycin was added to prevent infection and body temperature returned to normal 2 days later without using steroids and tocilizumab. No manifestations of neurotoxicity and other adverse effects in this patient. The time of neutrophil and platelet engraftment was 10 days and 11 days after ASCT, respectively. Cytokine monitoring after infusion of CART is shown in **Figure 1**. The patient achieved CR and has been in remission for four years. The follow-up is still ongoing. The treatment regimen of the patient is depicted in **Figure 2**.

**Figure 3:**
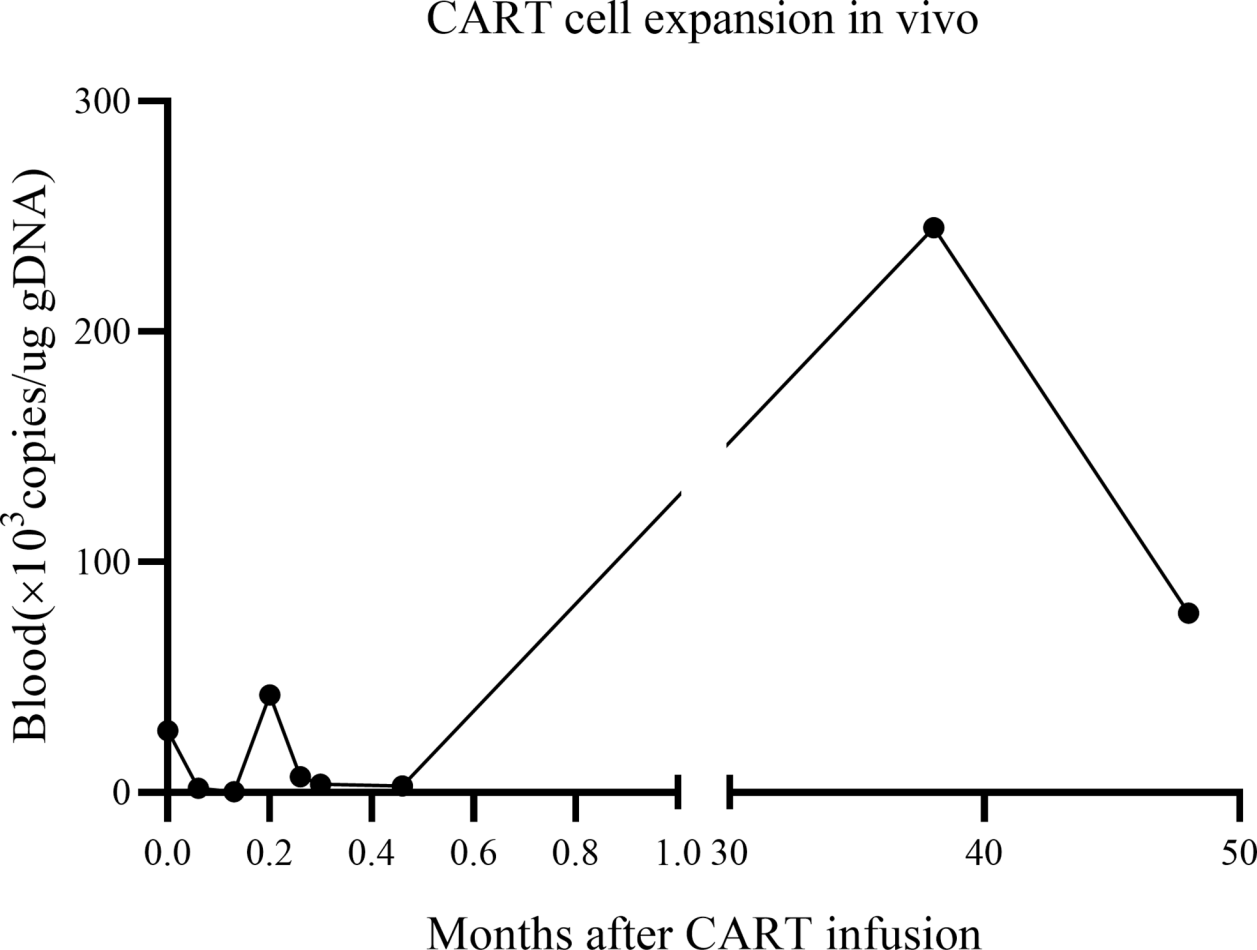
CART cell expansion *in vivo*. CART, chimeric antigen receptor T-cell.

The *in vivo* expansion and persistence of CART cells were monitored by reverse transcription polymerase chain reaction (**Figure 3**). CART cells reached their first peak 6 d after infusion and then dropped. The second peak was observed at 38 months after infusion, when the patient showed enlargement of the inguinal lymph nodes (the largest was 2.8 × 0.5 cm); moreover, the expression of *CAR* decreased as the lymph nodes shrunk to their normal size. At the follow-up 48 months after the patient’s CART infusion, the expression of *CAR* persisted *in vivo*, reaching to 2.55 × 10^5^ copies/ug gDNA in the bone marrow and 7.78 × 10^4^ copies/ug gDNA in the peripheral blood.

## Discussion

This case study is the first to report the treatment of a 61-year old, high-risk BL patient with ASCT combined with CART cell therapy and show that the patient achieved CR without any severe adverse events. This confirmed the safety and feasibility of using a combination of ASCT and CART cell therapy for BL. In addition, we observed long-term persistence of CART cells in the BM and peripheral blood. The effect of CART against BL is relatively unexplored (**Table 1**); therefore, our study provides novel insights into the potential applications of the therapy.

**Table 1:** Treatment and outcomes from prior trials of CART cell therapy for Burkitt lymphoma (Literature review).

Published online	Number of patients	Age(years)	IPI/Stage	Target	co-stimulatory domains	CRS	Response	HSCT	Survival months
18th, Jan., 2021 (9)	6	21-34	3 IPI4, 1 IPI3, 2 IPI1	CD19+CD22	CD28 and 4-1BB	5(I) 1(III)	1CR, 2PR, 1SD, 2PD	1	1-37^+ a^
25th, Jun., 2020 (10)	5	6-10	3 stageIV, 2 stageIII	3 CD19, 1 CD19+CD22, 1CD19+CD22+CD20	4-1BB	3(III), 2(I)	5CR	none	5^+^-14^+ b^
26th, Apr., 2018 (11)	1	32	StageIV	CD19	CD28	II	CR	1	0 ^c^
25th, Feb., 2021 (12)	1 ^d^	1.7	StageIV	CD19	4-1BB	II	CR	none	16^+^
20th, May., 2022 (13)	28	17-70	3 StageI-II, 25 StageIII-IV	CD19/CD22	CD28 and 4-1BB	16(I) 11(II-IV)	16CR 3PR 12PD	13	0-60^+^

^a^ The CR patient received allo-HSCT and is in ongoing remission at 37 months, the SD patient died after 4.5 months of disease progression, one of the PD patients died after a month of therapy, another three patients were enrolled into another clinical trial (CAR22/19-T cells following auto-HSCT), two of them showed no response to this clinical trial, whereas the third is under remission at 22 months.

^b^A median follow-up of 331 d, ranging from 149 to 428 days; all patients remained in CR.

^c^ Died of sepsis on the 9th day after HSCT.

^d^ A male patient was diagnosed with BL-PTLD after undergoing living liver transplantation.

IPI/Stage, international prognostic index or Ann Arbor stage; PTLD, post-transplant lymphoproliferative disorder; HSCT, hematopoietic stem cell transplantation.

Compared to the patients in previously reported cases of CART against BL, the patient in the present study was older. Our treatment plan may be a better choice to improve the low PFS rate in older patients who rely solely on chemotherapy. In previously reported cases, most patients required a second or even third round of CART treatment. However, in our case study, because the persisting CART cells helped prevent tumor relapse, the patient sustained CR and did not require any other further treatment.

The long-term persistence of CART cells might be attributed to two mechanisms. First, CART products in this patient showed a high proportion of naive(32.8%) and memory(44.7%) CART cells. Studies have shown that compared with T central memory cells, T memory stem cells from the CD45RA^+^ T cell population with high CD62L expression are more durable and effective against tumors (14). The naivety of CART cells can increase the effect of immunotherapy (15). In addition, the CART product in our study uses 4-1BB as the costimulatory domain. Compared to CD28 CART cells, 4-1BB CART cells show more memory phenotypes, express lower levels of depletion markers, and can retain effector functions for a long time under chronic antigen stimulation (16). Second, we believe that ASCT and its high dose transplantation conditioning decreased tumor burden, depleted the immunosuppressive microenvironment of lymphoma and deeply deplete regulatory T cells that inhibit CAR T-cell function,which enhanced CART cell persistence (17).

In summary, our novel combination of ASCT and CART therapy successfully cured a high-risk Burkitt lymphoma patient. In future, we will elucidate the mechanism behind persistence of CART cells and apply our treatment method to more high-risk Burkitt lymphoma patients to improve their prognosis.

## Data availability statement

The original contributions presented in the study are included in the article/supplementary material. Further inquiries can be directed to the corresponding author.

## Ethics statement

The studies involving human participants were reviewed and approved by the National Natural Science Foundation of China. The patients/participants provided their written informed consent to participate in this study.

## Author contributions

Treatment decision-making and discussions: LG, JYa. Data collection and analysis: MY, JYu, TW, and JG. Manuscript writing: MY and TW. Final approval of manuscript: JYa. All authors contributed to the article and approved the submitted version.

## Funding

We would like to acknowledge the funding support of the National Natural Science Foundation of China (Grant No. 81770209) and Shanghai 2021 “Action Plan of Technological Innovation” Biomedical Science and Technology Support Special Project (21S11906100).

## Acknowledgments

Firstly, I would like to extend my sincere gratitude to my supervisor, JMY, for his instructive advice and useful suggestions on my thesis and I am deeply grateful for his help in the completion of this thesis. I am also deeply indebted to all the other tutors and teachers in Translation Studies for both their direct and indirect help to me. Special thanks should go to my friends who have put considerable time and effort into their comments on the draft. Finally, I am indebted to my parents for their continuous support and encouragement.

## Conflict of interest

The authors declare that the research was conducted in the absence of any commercial or financial relationships that could be construed as a potential conflict of interest.

## Publisher’s note

All claims expressed in this article are solely those of the authors and do not necessarily represent those of their affiliated organizations, or those of the publisher, the editors and the reviewers. Any product that may be evaluated in this article, or claim that may be made by its manufacturer, is not guaranteed or endorsed by the publisher.

## Abbreviations

ASCT, Autologous stem cell transplantation; BL, Burkitt lymphoma or leukemia; CART, Chimeric antigen receptor T cells; CR, complete remission; IRB, Institutional review board; OS, Overall survival; PFS, Progression-free survival; VDCP, Vindesine, daunorubicin, cyclophosphamide, and dexamethasone.

## References

[1] Crombie J LaCasce A . The treatment of burkitt lymphoma in adults. Blood (2021) 137(6):743–50. doi: 10.1182/blood.2019004099 33171490

[2] Evens AM Danilov A Jagadeesh D Sperling A Kim SH Vaca R . Burkitt lymphoma in the modern era: real-world outcomes and prognostication across 30 US cancer centers. Blood (2021) 137(3):374–86. doi: 10.1182/blood.2020006926 PMC8765121 32663292

[3] Park JH Rivière I Gonen M Wang X Sénéchal B Curran KJ . Long-term follow-up of CD19 CAR therapy in acute lymphoblastic leukemia. N Engl J Med (2018) 378:449–59. doi: 10.1056/NEJMoal1709919 PMC6637939 29385376

[4] Maude SL Laetsch TW Buechner J Rives S Boyer M Bittencourt H . Tisagenlecleucel in children and young adults with b-cell lymphoblastic leukemia. N Engl J Med (2018) 378:439–48. doi: 10.1056/NEJMoal1709866 PMC5996391 29385370

[5] Schuster SJ Bishop MR Tam CS Waller EK Borchmann P McGuirk JP . Tisagenlecleucel in adult relapsed or refractory diffuse large b-cell lymphoma. N Engl J Med (2019) 380:45–56. doi: 10.1056/NEJMoal804980 30501490

[6] Schuster SJ Svoboda J Chong EA Nasta SD Mato AR Anak Ö . Chimeric antigen receptor T cells in refractory b-cell lymphomas. N Engl J Med (2017) 377:2545–54. doi: 10.1056/NEJMoa1708566 PMC5788566 29226764

[7] Locke FL Ghobadi A Jacobson CA Miklos DB Lekakis LJ Oluwole OO . Long-term safety and activity of axicabtagene ciloleucel in refractory large b-cell lymphoma (ZUMA-1): a single-arm, multicentre, phase 1–2 trial. Lancet Oncol (2019) 20:31–42. doi: 10.1016/S1470-2045(18)30864-7 30518502 PMC6733402

[8] Porter D Frey N Wood PA Weng Y Grupp SA . Grading of cytokine release syndrome associated with the CAR T cell therapy tisagenlecleucel. J Hematol Oncol (2018) 11:35. doi: 10.1186/s13045-018-0571-y 29499750 PMC5833070

[9] Zhou X Ge T Li T Huang L Cao Y Xiao Y . CAR19/22 T cell therapy in adult refractory burkitt’s lymphoma. Cancer Immunol Immunother (2021) 70:2379–84. doi: 10.1007/s00262-021-02850-6 PMC10991587 33459843

[10] Zhang W Yang J Zhou C Hu B Jin L Deng B . Early response observed in pediatric patients with relapsed/refractory burkitt lymphoma treated with chimeric antigen receptor T cells. Blood (2020) 135(26):2425–27. doi: 10.1182/blood.2019002008 32321169

[11] Avigdor A Shouval R Jacoby E Davidson T Shimoni A Besser M . CAR T cells induce a complete response in refractory burkitt lymphoma. Bone Marrow Transplant (2018) 53:1583–85. doi: 10.1038/s41409-018-0235-0 29795432

[12] Wang T Feng M Luo C Wan X Pan C Tang J . Successful treatment of pediatric refractory burkitt lymphoma PTLD after liver transplantation using anti-CD19 chimeric antigen receptor T-cell therapy. Cell Transplant (2021) 30:963689721996649. doi: 10.1177/0963689721996649 33631963 PMC7917414

[13] Jiaying Wu Yang C Qi Z Wanying L Xiaoxi Z Xi M . Chimeric antigen receptor-modified T cell immunotherapy for relapsed and refractory adult burkitt lymphoma. Front Immunol (2022) 13:879983. doi: 10.3389/fimmu.2022.879983 35669773 PMC9164136

[14] Gattinoni L Klebanoff CA Restifo NP . Paths to stemness: building the ultimate antitumour T cell. Nat Rev Cancer (2012) 12:671–84. doi: 10.1038/nrc3322 PMC6352980 22996603

[15] Golubovskaya V Wu L . Different subsets of T cells, memory, effector functions, and CAR-T immunotherapy. Cancers (2016) 8(3):36. doi: 10.3390/cancers8030036 PMC4810120 26999211

[16] Sievers NM Dörrie J Schaft N . CARs: beyond T cells and T cell-derived signaling domains. Int J Mol Sci (2020) 21(10):3525. doi: 10.3390/ijms21103525 PMC7279007 32429316

[17] Wang T Gao L Wang Y Zhu W Xu L Wang Y . Hematopoietic stem cell transplantation and chimeric antigen receptor T cell for relapsed or refractory diffuse large b-cell lymphoma. Immunotherapy (2020) 12(13):997–1006. doi: 10.2217/imt-2020-0075 PMC7546158 32752910

